# Refractive Outcomes after Cataract Surgery

**DOI:** 10.3390/diagnostics12020243

**Published:** 2022-01-19

**Authors:** Ramin Khoramnia, Gerd Auffarth, Grzegorz Łabuz, George Pettit, Rajaraman Suryakumar

**Affiliations:** 1 The David J Apple International Laboratory for Ocular Pathology, Department of Ophthalmology, University Hospital Heidelberg, Im Neuenheimer Feld 400, 69120 Heidelberg, Germany; Gerd.Auffarth@med.uni-heidelberg.de (G.A.); Grzegorz.Labuz@med.uni-heidelberg.de (G.Ł.); 2 Alcon Research LLC, Fort Worth, TX 76134, USA; george.pettit@Alcon.com (G.P.); rajaraman.suryakumar@Alcon.com (R.S.)

**Keywords:** biometry, refractive outcomes, cataract surgery, intraocular lenses, prediction accuracy, visual acuity

## Abstract

A post-operative manifest refractive error as close as possible to target is key when performing cataract surgery with intraocular lens (IOL) implantation, given that residual astigmatism and refractive errors negatively impact patients’ vision and satisfaction. This review explores refractive outcomes prior to modern biometry; advances in biometry and its impact on patients’ vision and refractive outcomes after cataract surgery; key factors that affect prediction accuracy; and residual refractive errors and the impact on visual outcomes. There are numerous pre-, intra-, and post-operative factors that can influence refractive outcomes after cataract surgery, leaving surgeons with a small “error budget” (i.e., the source and sum of all influencing factors). To mitigate these factors, precise measurement and correct application of ocular biometric data are required. With advances in optical biometry, prediction of patient post-operative refractory status has become more accurate, leading to an increased proportion of patients achieving their target refraction. Alongside improvements in biometry, advancements in microsurgical techniques, new IOL technologies, and enhancements to IOL power calculations have also positively impacted patients’ refractory status after cataract surgery.

## 1. Introduction

Cataract surgery with intraocular lens (IOL) implantation is one of the most common ophthalmic procedures in clinical practice; however, post-operative refractive outcomes remain a key area of concern for surgeons [1]. Improvements in surgical technique, new IOL technologies, enhanced biometric methods, and advanced methods of IOL power calculation have led to modern cataract surgery being a refined procedure that enables most patients to achieve high-quality post-operative vision [1]. In line with advances in cataract surgery, there is an increasing patient expectation of excellent post-operative outcomes and high demand for spectacle independence, particularly in developed countries [1,2,3]. However, unsatisfactory visual outcomes due to residual refractive errors may occur and remain an important cause of visual disability and poor quality of life [1,2,3]. For example, refractive surprise (i.e., any deviation from intended target) after cataract surgery, is unsatisfactory for both the patient and the surgeon, and correction of large refractive errors requires additional procedures [1]. There are a plethora of pre-, intra-, and post-operative factors that can influence refractive outcomes, leaving surgeons with little room for error (i.e., a small “error budget”): for this reason, a high level of diagnostic precision, including meticulous and accurate collection of measurements and appropriate application of ocular biometric data, is required.

In this review, we will examine key factors that may affect error prediction accuracy, and how residual refractive error impacts visual outcomes. We will also discuss how refractive outcomes have improved with the evolution of ocular biometry, and how modern technologies have positively impacted patients’ refractory status following cataract surgery.

## 2. Refractive Outcomes Prior to the Advent of Modern Optical Biometry

The first great advancement in ocular biometry was the development of ultrasound A-scan biometry, first described in the 1960s as a method that allowed visualization along an ultrasonic path to ensure alignment with a patient’s visual axis [4]. Ultrasound A-scan biometry values are obtained either by immersion or applanation methods: the immersion technique places a saline-filled scleral shell between the patient’s eye and an ultrasound probe, whereas the applanation method requires placing the ultrasound probe directly on the central cornea [5]. Prospective studies have demonstrated that the immersion technique has better reliability and fewer post-operative refractive errors compared with the applanation method [5,6]. Mean ± standard deviation axial lengths (AL) measured using applanation A-scan biometry can vary by 0.14 ± 0.12 mm compared with 0.03 ± 0.04 mm for immersion [5]. In addition, AL acquired by applanation can be 0.1–0.3 mm shorter than AL acquired by immersion [6,7]. The greater variability of AL measurements with the applanation technique could translate into larger post-operative refractive errors, as was demonstrated in a prospective, randomized trial of 288 patients in which the mean absolute prediction error (MAE) was 0.53 ± 0.48 diopters (D) for the applanation group and 0.43 ± 0.38 D for the immersion group [6]. From the safety outcomes perspective, direct contact of an ultrasound probe with the patient’s eye not only requires topical anesthesia [8], but also risks corneal epithelial injury, infection, and causes patient discomfort [9]. In terms of refractive outcomes, direct contact with the central cornea risks creating corneal compressions that may introduce errors in the biometric readings acquired during applanation A-scan biometry [5,6], in turn leading to errors in the predicted refraction [6]. Despite these drawbacks, applanation A-scan biometry was widely used and, together with immersion A-scan biometry, the introduction of biometric techniques greatly improved refractive outcomes [10,11].

**Figure 1:**
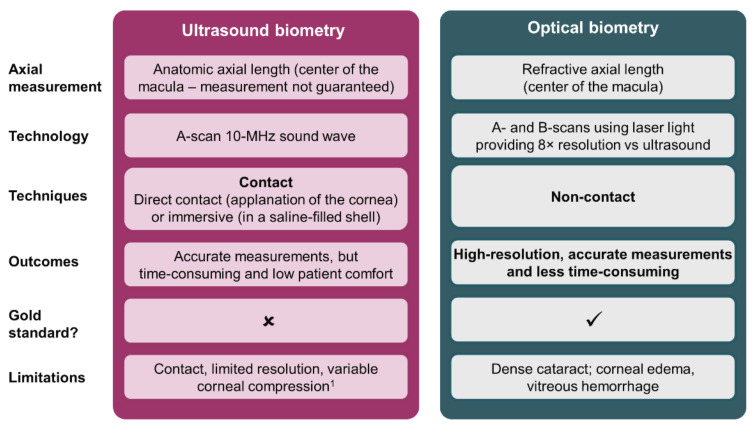
Overview of ultrasound and optical biometry techniques [5,8,9,17,18,19]. ^1^ Only applicable to applanation biometry, corneal compression is not observed in immersion biometry.

In the late 1990s, technologic advances in biometry continued with the introduction of optical biometry—a technique based on partial coherence interferometry (PCI) [12]. Compared with ultrasound biometry, which uses a 10-MHz sound wave [5], optical biometry uses infrared light technology [13], offering enhanced resolution (~12 μm) and more than 10-times greater precision (<10 μm) [12,14]. As a non-contact technique, optical biometry is less time-consuming than ultrasound biometry because it does not require local anesthesia or pupil dilation, in turn reducing patient discomfort (Figure 1) [8,12]. In addition, the non-contact approach eliminates variations due to corneal compressions, as is commonly seen with applanation A-scan biometry [9]. These advantages translate into better post-operative refractive outcomes when compared with applanation [8,12,15] and immersion biometry [16]. Prospective, randomized studies have shown that patients who underwent optical biometry using PCI achieved an MAE of 0.30–0.52 D compared with 0.62–0.94 D with applanation biometry [8,15]. Within these studies, 87–100% of patients in the PCI groups and 71–80% of patients in the applanation groups were within ±1.0 D of their target refraction [8,15]. In addition, 93% of patients in the PCI group and 85% of patients in the immersion group were within ±1.0 D of their target refraction (*p* = 0.04) [16].

The impact of optical biometry on refractive outcomes after cataract surgery can be appreciated by observing the steadily increasing percentage of patients achieving within ±1.0 D or ±0.5 D of their target refraction in studies between 1992 and 2017 [10,11,20,21,22,23,24]. Between 1992 and 2006, studies showed that 72.3–87.0% of patients achieved a deviation from the target refraction of ±1.0 D [10,11,20,21], which increased to 89.6–97.3% between 2007 and 2017 [22,23,24]. Between 1996 and 2005, studies showed that 44.6–58.4% of patients achieved a deviation from the target refraction of ±0.5 D [10,11], a proportion that increased to 61.2–88.0% between 2007 and 2017 [22,23,24,25,26]. It is therefore evident that, since its introduction in the 1980s [12], optical biometry using PCI has considerably improved refractive outcomes in clinical practice (Table 1) [11]. The magnitude of these improvements is, perhaps, best described by results from a prospective, multicenter, comparative, non-randomized study of 23,244 patients in the Swedish National Cataract Register that reported the outcomes of cataract extraction performed from 2000 through 2005 [11]. This study showed that there was a significant difference in MAE over 6 years (*p* < 0.0001), with the MAE consistently reducing between 2000 (~0.67 D) and 2004 (~0.51 D), likely driven by the gradual uptake of optical biometers in ophthalmology departments [11].

Innovations in optical biometry technology have continued to emerge with the introduction of biometers that use swept-source optical coherence tomography (SS-OCT), such as IOLMaster 700 (Carl Zeiss Meditec AG, Jena, Germany), ARGOS (Movu, Inc., San José, CA, USA), and OA-2000 (Tomey, Nagoya, Japan) [27]. SS-OCT is a non-invasive, high-speed method that collects thousands of scans from an extended imaging axial range within 1 second and generates 2- or 3-dimensional data with high lateral resolution and axial resolution [28]. This principle is at the core of the ARGOS, IOLMaster 700, and OA-2000 biometers, which use a ~1060-nm wavelength swept-source technology [28]. SS-OCT biometers provide OCT images of the entire eye by scanning up to 2000 times per second and providing measurements of AL, anterior chamber depth (ACD), central corneal thickness, lens thickness, aqueous depth, pupil size, corneal diameter, and keratometry [27,28]. SS-OCT biometers utilize either composite or segmented methods to obtain measurements of the eye, of which segmented analysis has been shown to be more accurate than composite analysis for eyes with long AL [29]. Furthermore, the longer wavelength utilized by SS-OCT biometers can penetrate dense cataracts, thereby providing accurate measurements for a broad range of patients [27,30].

## 3. Pre-, Intra-, and Post-Operative Factors That Affect Refractive Outcomes

In this section, we review the main factors that affect current refractive outcomes.

### 3.1. Pre-Operative Factors

**Figure 2:**
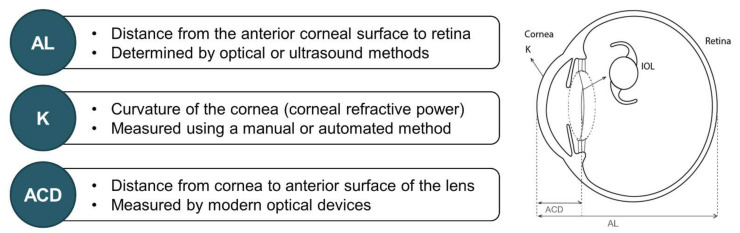
Key biometric parameters involved in IOL power calculation [2,3,31,32,33,34,35]. ACD = anterior chamber depth; AL = axial length; IOL = intraocular lens; K = keratometry.

Pre-operative biometric data, derived from measuring the physical dimensions of the eye, are necessary to determine the refractive power of the IOL to be implanted [2,31]. Key biometric parameters involved in IOL power calculations, including AL, corneal power, and ACD, are detailed in the following sections and outlined in Figure 2.

#### 3.1.1. Axial Length

AL measurement is one of the most critical steps in IOL power calculation: this parameter should ideally be accurate within 0.1 mm because such a small error equates to a post-operative refraction error of ~0.27 D [33]. Measurements of AL using SS-OCT are more accurate than ultrasound biometry, with a median accuracy of 0.05 mm and 0.12 mm, respectively [36].

#### 3.1.2. Corneal Power

Corneal power is another important measure to optimize refractive outcomes, as keratometric errors of 0.5 D in corneal power can lead to an error of 0.5 D in post-operative refraction [3]. Keratometry can be performed using manual or automated methods: the manual method takes four measurements 3.2 mm from the corneal center; whereas the automated method takes four to six radial measurements 2.6 mm from the center of the cornea [3]. Keratometry has two limitations: first, it measures only the radius of the anterior corneal surface’s curvature and considers a population average effect, when in actuality, the posterior corneal surface/aqueous interface contributes uniquely to the net corneal power. In addition, irregular corneas do not have a consistent anterior surface curvature; therefore, detailed topography (>5000 points of measurement) must be employed in such cases, to characterize overall refractive power [3,37].

#### 3.1.3. Pre-Operative Corneal Astigmatism

Pre-operative corneal astigmatism is a very common condition and can lead to residual refractive errors after cataract surgery [1,3]. Corneal astigmatism of ≥0.5 D occurs in approximately 78% of eyes (based on real-world assessment of 110,468 eyes) and ≥1.0 D in approximately 42% of eyes [38]. Corneal astigmatism can be classified based on the location of the steepest meridian—in eyes that have with-the-rule (WTR) astigmatism the vertical meridian is steepest and conversely, the horizontal meridian is steepest in against-the-rule (ATR) astigmatism [39]. Corneal astigmatism can be corrected by the placement of incision on the steeper axis, peripheral corneal relaxing incisions, and use of a toric IOL [3]. A retrospective analysis of corneal topography data from 641 eyes concluded that the magnitude of corneal astigmatism decreases from the center to the mid-periphery, and the extent of the difference depends on the size and the type of corneal astigmatism [40]. As a change in corneal power from the center to the periphery of the cornea could potentially lead to suboptimal refractive correction in some cases, as indicated by ray-tracing simulations, more research is required to assess the impact of mid-peripheral astigmatism on patients’ visual function [40,41].

Mounting evidence suggests that posterior corneal astigmatism is clinically relevant when assessing the effect of astigmatism on refractive outcomes. In a prospective, observational study of 493 patients, posterior corneal astigmatism reduced total corneal astigmatism by approximately 13% and total corneal astigmatism differed from anterior corneal astigmatism by >0.5 D in around one-third of eyes [42]. In a case series that analyzed 715 eyes, the posterior cornea was steeper along the vertical meridian in >80% of eyes and, being a minus lens, caused a plus refractive power horizontally (ATR refractive astigmatism) [43]. Additionally, the level of posterior astigmatism varied according to the level of anterior corneal astigmatism—approximately 0.5 D in corneas that had WTR anterior astigmatism and 0.3 D for those with ATR anterior astigmatism [43,44]. Although complex, the relationship between anterior and posterior corneal astigmatism follows distinct trends: the posterior cornea tends to partly compensate for increasing amounts of WTR anterior astigmatism, and is relatively constant in eyes with increasing amounts of ATR astigmatism [43]. Overall, calculating posterior corneal power based on a fixed ratio between anterior and posterior curvatures may introduce errors of up to 0.5 D [39].

#### 3.1.4. Anterior Chamber Depth

Even though ACD measurement is required to increase the accuracy of the IOL power prediction curve with modern formulae [3], incorrect assessment of this parameter is the largest source of refractive error [45]. An estimated 1-mm error in post-operative ACD equates to a refractive error of 1.44 D for regular eyes [45]. It is worth noting that different definitions of ACD exist within the literature depending on the biometer used: for example, some biometers measure ACD from the corneal endothelium to the anterior lens surface, others from the corneal epithelium to the anterior lens surface [3,46].

#### 3.1.5. IOL Power Calculations

Calculation of the IOL power is fundamental to enable patients to achieve desired refractive outcomes [32]. Accurate IOL power calculations rely on numerous factors, such as accurate pre-operative biometric measurements, precise prediction of effective lens position (ELP), appropriate IOL formula selection, and optimization of the IOL constant [2,45]. No single formula is suitable for all eyes, and each formula requires a different selection of biometric data [3]. Even though IOL constants provided by manufacturers allow for an initial estimation of best-suited IOL power [47], optimization is required to minimize systematic errors, and may also improve the accuracy of IOLs manufacturing [48].

#### 3.1.6. Other Pre-Operative Considerations

Previous corneal refractive surgery should be considered when obtaining biometric measurements and selecting the appropriate IOL formula because keratometric values change after surgery [3]. Inaccurate biometric measurements and inappropriate IOL formula selection may lead to refractive surprise: hyperopic surprise often occurs in patients with previous myopic correction, and myopic surprise in patients with previous hyperopic correction [49]. Conversely, good refractive outcomes can be achieved, even in complicated cases, if a customized approach to biometric assessment and IOL implantation is used [50].

### 3.2. Intra-Operative Factors

Surgical technique is the key intra-operative factor that may influence refractive outcomes after cataract surgery. Indeed, predictability of refractive outcomes has improved because of refinements in surgical procedures [25]. Optimal refractive outcomes can be achieved by employing good surgical technique, aiming for a low rate of posterior capsular rupture, having a capsulorhexis size smaller than the optical diameter, and having an in-the-bag IOL placement [2]. Continuous curvilinear capsulorhexis ensures positional stability and enhances refractive predictability [2,25], while small incisions without sutures and the use of foldable IOLs reduce the incidence of complications and surgically induced astigmatism (SIA) [51], and complete ophthalmic viscosurgical device removal reduces the likelihood of IOL misalignment [52]. Non-intentional SIA can be prevented by pre-operative assessment of corneal hysteresis and biomechanical properties of the cornea, followed by microincision surgery using corneal topography data and standard IOL power formulae [3]. The magnitude of SIA depends on the incision size and site during surgery [53,54]. Small incision sizes of 2.0–2.2 mm are recommended with increasing frequency as IOL delivery methods advance [55,56]. However, results from a prospective study of 58 eyes undergoing IOL implantation found that the final wound size was ~2.4 mm due to wound stretch during implantation, irrespective of the incision size prior to implantation [57]. This wound enlargement might, in some cases, be caused by injector nozzle tip damage during the implantation process [58]. Additionally, eyes with greater incision enlargement tended to have higher SIA, suggesting that a clean corneal incision prior to implantation may be preferential to stretching the corneal incision [57].

### 3.3. Post-Operative Factors

Post-operatively, the astigmatism-correction power of toric IOLs may be reduced as a consequence of off-axis rotation [59]: a 1° rotation results in 3.3% loss of astigmatism correction, and a ≥30° rotation leads to no reduction in astigmatism magnitude but a large change in axis [60]. ELP, that is, the position of the IOL in the eye (specifically, the distance between the principal object plane of the IOL and the principal image plane of the cornea) [61] differs for each IOL design and displacement can significantly affect refraction and IOL power predictions [62,63]. Forward deviation of an IOL leads to myopia, and conversely backward deviation leads to hyperopia [62]. Decentration or tilt may also affect post-operative refractive errors by inducing increased astigmatism and coma aberrations [64,65]. In an optical bench study, it was found that aspheric monofocal lenses were less negatively affected by decentration than aspheric diffractive bifocal or trifocal lenses, with mean optical quality reduction of <10% for 1-mm decentration at physiologic pupil sizes. The optical quality at all distances for diffractive bifocal and trifocal lenses was significantly reduced if decentration was more than 0.75 mm, with intermediate focus showing the least reduction [66]. In a prospective, non-comparative case series, monofocal anterior capsulotomy-fixated IOLs had low levels of decentration and high in-the-bag stability over a 1-year period [67].

By examining refractive accuracy in cataract surgery, the post-operative refraction measurement itself is also a source of error. Multiple studies indicate that the 95% test-retest spherical equivalent refraction measurement is approximately ±0.5 D [68,69,70,71,72]. This means that even if all other error sources are eliminated, it is highly unlikely that 100% of eyes would achieve outcomes within 0.5 D of the intended target.

Despite these limitations, retrospective analyses suggest that for patients with substantial interocular anatomic symmetry (such as patients without prior monocular refractive surgery or certain pathologic conditions) first-eye refractive results can be used to refine the treatment plan for the second eye and improve outcomes [73,74].

## 4. How Modern-Day Biometry Has Changed Refractive Outcomes

Modern-day optical biometry has improved refractive outcomes in several ways. A key example is represented by optical biometers, which allow a high success rate of AL measurements, ranging from 77.0–88.4% with PCI biometers, 79.0% with optical low-coherence reflectometry biometers, to 92.6–99.4% with SS-OCT biometers, with the best results to date reported using ARGOS (96.0–99.4%) [28,75,76,77,78]. Additionally, SS-OCT optical biometers allow for a high degree of repeatability and reproducibility for most biometric measurements and are compatible with a wide range of IOL power calculation formulae, resulting in good refractive outcomes [79,80,81]. SS-OCT biometers demonstrate excellent precision across a range of measurements, including AL, ACD, lens thickness, and anterior corneal radius of curvature [28,76] with low coefficient of variation and high intraclass correlation coefficient [76,77]. In instances where acquisition cannot be achieved with an optical biometer, such as dense cataracts, the enhanced retinal visualization mode of SS-OCT biometers can be utilized instead of ultrasound biometry: by using this mode, the sensitivity at the retina is enhanced by 10-times. In eyes with dense cataracts, ARGOS has proved successful at measuring AL [27,28] and AL acquisition rates are significantly higher with ARGOS (89.9%) and OA-2000 (80.8%) compared with IOLMaster 700 (63.6%) in eyes with grade IV cataract or higher [27]. With modern biometry, a prediction error of ±0.25 D can be achieved in 40–52% of patients, depending on the IOL formula used [80]. Measuring AL using multiple indices of refraction specific to each component of the eye increases the proportion of patients achieving ±0.5 D of the target refraction compared with a PCI optical biometer by up to 13.9% [82].

## 5. The Impact of Residual Refractive Error on Visual Outcomes

Results from patient-reported outcomes/satisfaction questionnaires have shown that a large proportion of patients (73–98%) were satisfied overall with their vision following cataract surgery and multifocal IOL implantation [83,84,85]. Nonetheless, a large proportion of patients reported glare, halos, and starburst symptoms (13–85%), and achievement of complete spectacle independence varied widely (31–95%) [83,84,85,86]. It is worth noting that the questionnaires used to evaluate patient satisfaction and reporting of the results vary within the literature and caution should be exercised when interpreting the outcomes and comparing such studies. Refractive errors are one of the main causes of poor vision, reported among 11–42% of patients assessed in population-based studies [87,88,89]. Residual refractive errors after cataract surgery can negatively impact patients’ uncorrected near, intermediate, and distance vision: in general, the larger the refractive error, the worse the patient’s vision [90,91,92,93]. Patients may experience blurred vision, with or without photic phenomena, as well as problems with reading in mesopic conditions following IOL implantation, ultimately impacting their quality of life, which can lead to their dissatisfaction [94].

Residual refractive errors can be adjusted with glasses or contact lenses [3]. Refractive surprise involving large errors in spherical or cylindrical power can be corrected with corneal-based laser refractive surgery [3] or lens-based procedures [1,3,95], each of which has advantages and limitations. Advantages of corneal-based laser refractive surgery are that additional intraocular surgery can be avoided, spherical and astigmatic refractive errors can be corrected [3]. However, drawbacks include the low availability of excimer lasers, high comparative cost, and the fact that correction of high residual refractive error depends on corneal thickness [3]. Lens-based procedures, such as in situ fine-tuning of light-adjustable IOLs, piggyback IOLs, supplementary IOLs, and IOL exchange can be used to correct large refractive errors where excimer lasers are not available [3]. In situ fine-tuning of light-adjustable IOLs is non-invasive, can be personalized to patient requirements and preferences, can adjust up to 2.0 D of sphere and cylinder in one procedure, and it is performed after complete healing has taken place and the IOL is locked in position (which increases refractive stability over time) [1,95,96]. This method has several disadvantages, some of which may result in additional visits to the ophthalmologist’s office (such as potential risk of macular burn due to ultraviolet light-based technology, requirement of pupil dilation of at least 6.5 mm, and repeat adjustments, which can also lead to dilation fatigue); in addition, it can only be performed on specific light-adjustable IOLs [95,97]. Piggyback and IOL exchange can correct large residual refractive spherical errors, do not change the corneal refractive power, and can be implanted via the original incision soon after initial surgery [3]. The development of an interface membrane between the IOLs is a disadvantage of piggyback IOL, and lens exchange can cause SIA due to wound enlargement while removing the original IOL [3]. Supplementary IOLs are designed for implantation into the ciliary sulcus and can be used to correct post-operative refractive errors without the requirement to exchange the IOL implanted into the capsular bag [98]. Ciliary sulcus-fixated IOLs can be prone to decentration or tilting, leading to decreased image quality and complications from ciliary-body contact. However, case reports suggest that supplementary IOLs have a high tolerance to misalignment and minimal light attenuation and can easily be removed or exchanged from the ciliary sulcus [98,99,100,101]. The impact of residual astigmatism could also be modestly influenced by the optics of the IOL. A recent study reported that diffractive extended depth of focus IOLs showed statistically significantly better uncorrected visual acuity (VA) compared with monofocal control; however, these differences were modest at best (1–2 letters for manifest refractive spherical equivalents within 1.0 D between groups and approaching borderline statistical significance) and hence, it is difficult to conclusively report if they are clinically meaningful [93]. Some studies that have conducted astigmatic defocus curve testing have shown that extended depth of focus IOLs may have a greater tolerance to residual refractive errors compared with trifocal and bifocal IOLs [102,103]. However, this type of testing is not straightforward and most studies in the literature do not maintain spherical equivalent when increasing astigmatic errors by more than 0.5 D and overall effect from different cylinder axis positions between manifest refraction and induced cylinder are also not addressed. Hence, a standardized way of conducting such tests is lacking.

The relationship between refractive error and uncorrected visual acuity (UCVA) is not straightforward, in the fact that different types of refractive error contribute differently to vision loss—for example, deterioration in distance VA is greater with myopic astigmatism (0.31 logarithm of the minimum angle of resolution [logMAR] per D of astigmatism) than with hyperopic astigmatism (0.23 logMAR per D of astigmatism) [104]. Changes in vision can be inferred from the changes in the magnitude of blur, which can be quantified using a blur strength metric that includes spherical equivalent, horizontal/vertical astigmatism, and oblique astigmatism [104].

In an interventional case series of 493 eyes undergoing unilateral cataract surgery and implantation of monofocal IOLs, it was found that uncorrected distance visual acuity (UDVA) worsened with increasing magnitude of myopic refractive error, was optimal at emmetropia, and worsened with increasing hyperopic refractive error. UDVA and uncorrected near visual acuity (UNVA) intersected in the refractive error range of −1.0 to −1.5 D. The fact that less than half of patients (37%) achieved a post-operative corrected distance visual acuity (CDVA) of 20/20 Snellen or better, a UDVA of 20/32 Snellen, and a UNVA of Jaeger 4 suggests that satisfactory UCVA cannot always be obtained for both distance and near vision with implantation of monofocal IOLs [92]. For patients requiring full spectacle independence, trifocal IOLs may therefore represent a more appropriate choice [105,106,107,108].

It is also important to accurately report the impact of residual refractive error on VA. Snellen and logMAR are two of the most commonly used charts for assessing VA [109,110]; however, as most logMAR charts have a “bottom line” of −0.3 logMAR and use a 0.1 logMAR progression of letter size, whereas Snellen charts are usually truncated at 6/5 or 20/15 and have irregular progression in letter size, transposing Snellen directly into logMAR is not considered accurate and caution should be exercised when converting data from Snellen charts into logMAR [110]. LogMAR charts, namely the Early Treatment Diabetic Retinopathy Study (ETDRS) chart, are widely used in clinical trials as they are generally accepted as the more accurate, sensitive, and reproducible test [109,110], and should therefore be favored over Snellen when reporting the impact of residual refractive errors on VA.

## 6. Conclusions

The main goals of modern cataract surgery are rehabilitation of patients’ vision and achievement of on-target refraction. Post-operative refractive errors and residual astigmatism negatively impact VA and patient satisfaction, and therefore are of key concern to cataract surgeons. Various pre-, intra-, and post-operative factors influence refractive outcomes after cataract surgery, and accurate assessment and optimization of these factors are essential to achieving desired vision. The introduction of ultrasound biometry in the 1960s greatly improved refractive outcomes and, since then, biometric technology has continued to evolve. Optical biometry offered enhanced resolution and greater precision, and the non-contact method eliminated inaccuracies due to corneal compression, in addition to being more comfortable for patients. With further refinements in optical biometry, the proportion of patients achieving their target refraction has steadily increased. Innovations in optical biometry have continued to emerge with the introduction of biometers that utilize SS-OCT. Current research is advancing OCT methods further, for example through utilization of a large number of optical probe beams simultaneously (the “hyper-parallel” approach) to capture accurate anatomical snapshots with no eye motion degradation. Improvements such as these, alongside advancements in microsurgical techniques, new IOL technologies, and enhancements to IOL power calculations, continue to positively impact patients’ refractory status after cataract surgery.

## Figures and Tables

**Table 1:** Target refraction prediction errors across various studies.

	Study	Surgical Period	Data Source	N	Follow-Up Time	PE (%)		
						≤±0.5 D	≤±1.0 D	>1.0 D
Studies published prior to 2010	Lundstrӧm et al., 2002 [21]	1992–2000	Swedish National Cataract Register	405,149 ^1^	-	-	79.2	13–28%
	Murphy et al., 2002 [10]	1996–1999	Teaching hospital, England	1676 ^2^	3 weeks ^3^	44.6	72.3	
	Kugelberg and Lundstrӧm, 2008 [11]	2000–2005	Swedish National Cataract Register	23,244 ^4^	-	58.4	83.8	
	Gale et al., 2009 [20]	2003–2006	Single NHS center, England	4806 ^5^	~4 weeks	-	79.7–87.0	
Studies published after 2010	Hahn et al., 2011 [23]	2007–2008	Seven private practices, Germany	1553 ^1^	3 months	80.3	97.3	3–10%
	Jivrajka et al., 2012 [26]	2010	Single center, USA	250 ^2,4^	6–8 weeks	61.2	89.6	
	Aristodemou et al., 2011 [25]	2005–2010	Single NHS center, England	1867 ^4^	≥4 weeks	74.5	95.9	
	Behndig et al., 2012 [22]	2008–2010	Swedish National Cataract Register	17,056 ^2^	1–2 months	71.4	92.7	

D = diopter; NHS = National Health Service; PE = prediction error. ^1^ Number of interventions; ^2^ Number of eyes; ^3^ Or when all sutures removed and no further treatment planned; ^4^ Number of patients; ^5^ Number of datasets.
